# Relationship between humeral head translation, spur formations, and the locations of tendon tears in massive rotator cuff tears

**DOI:** 10.1186/s13104-024-07079-x

**Published:** 2025-01-27

**Authors:** Yuki Yoshida, Atsushi Yoshida

**Affiliations:** 1 Department of Orthopaedic Surgery, Fussa Hospital, 1-6-1 Kamidaira, Fussa, Tokyo, 197-8511 Japan; 2 Department of Orthopaedic Surgery, Chiba GEKA-NAIKA Hospital, 4-41 Haramachi, Kawaguchi, Saitama 332-0025 Japan

**Keywords:** Massive rotator cuff tear, 3DCT, Humeral migration, Humeral translation, Coracoacromial spur, Imaging

## Abstract

**Objective:**

This study aimed to clarify the relationship between the directions of humeral head translation, the presence of acromial or coracoid spurs, and the locations of tendon tears in massive rotator cuff tears. Thirty shoulders from thirty patients with massive rotator cuff tears who underwent reverse shoulder arthroplasty were included. Preoperative 3DCT classified humeral head translation into three groups: minimal type, posterosuperior type, and anterosuperior type. The presence of acromial or coracoid spurs was also assessed. Preoperative MRI and intraoperative findings determined the torn tendons in each rotator cuff, along with ruptures of the anterior fascia covering the subscapularis or the long head of the biceps tendon (LHB). Relationships between humeral head translations, spur formations, and tendon tear locations were analyzed using chi-square tests and adjusted standardized residuals.

**Results:**

Acromial spurs were more frequent in the posterosuperior type, while coracoid spurs, subscapularis tears, anterior fascia ruptures, and LHB ruptures were significantly associated with the anterosuperior type. Anterior fascia ruptures were significantly less frequent in the minimal type. Anterosuperior humeral head translation and coracoid spurs indicate subscapularis tears, anterior fascia ruptures, and LHB ruptures.

## Introduction

The rotator cuff muscles are responsible for stabilizing and moving the shoulder [1], and tears in these muscles are a common cause of shoulder pain and changes in anatomical position [2]. Given the various configurations of rotator cuff tears, dysfunction frequently accompanies these tears. Therefore, it is important to evaluate the anatomical positioning changes associated with rotator cuff tears.

The acromiohumeral distance (AHD) is widely recognized as a diagnostic criterion for predicting the presence of a rotator cuff tear [3, 4]. In the case of tears, the AHD narrows due to superior translation of the humeral head [5, 6], and an acromial spur is often observed [7]. Narrowing of the coracohumeral distance (CHD) and the presence of a coracoidal spur also imply a rotator cuff tear [8]. The various configurations of humeral head translation depend on the location of the tears.

Three-dimensional computed tomography (3DCT) is a valuable tool for evaluating humeral head translation and has been used in previous studies [9–12]. However, the relationship between the directions of humeral head translation, the presence of acromial or coracoidal spurs, and the locations of tendon tears has not been fully explored.

Massive rotator cuff tears, which involve two or more tendons [13], can lead to significant humeral head translation and spur formation, resulting in shoulder dysfunction. In patients with irreparable massive rotator cuff tears who undergo reverse shoulder arthroplasty, 3DCT and magnetic resonance imaging (MRI) are performed preoperatively to evaluate humeral head translations, spur formations, and rotator cuff tears. Additionally, the precise location of tendon tears can be identified intraoperatively. This study aimed to clarify the relationship between the directions of humeral head translation, the presence of acromial or coracoidal spurs, and the locations of tendon tears in patients with massive rotator cuff tears who underwent reverse shoulder arthroplasty.

## Materials and methods

### Participants

This retrospective case-control study evaluated patients with massive rotator cuff tears who underwent reverse shoulder arthroplasty between 2018 and 2021. Patients were included if they underwent preoperative CT and MRI scans, with the locations of tendon tears confirmed intraoperatively. Those who underwent reverse shoulder arthroplasty for primary osteoarthritis, rheumatoid arthritis, or proximal humeral fractures were excluded.

A total of 30 shoulders from 30 patients (15 males and 15 female) (mean age 76.5 ± 5.4 years) with massive rotator cuff tear who underwent revers shoulder arthroplasty were enrolled in this study. Their massive rotator cuff tears were defined as being greater than 5 cm [14] and involving a complete tear of at least two tendons [13]. Additionally, their supraspinatus tendon exhibited severe fatty degeneration, classified as Goutallier stage 4. Their preoperative active range of motion was severely limited, characterized by pseudoparalysis with less than 90° of active elevation, despite having no neurologic impairment [15].

### Image acquisition

Preoperative CT scans were performed for each participant with their arms in a neutral position. The CT data were collected in Digital Imaging and Communication in Medicine format, and 3D CT surface models of the scapula and humerus were reconstructed using AVIZO software (version 9.3.0; Maxnet, Tokyo, Japan).

### Humeral head translations

**Fig. 1:**
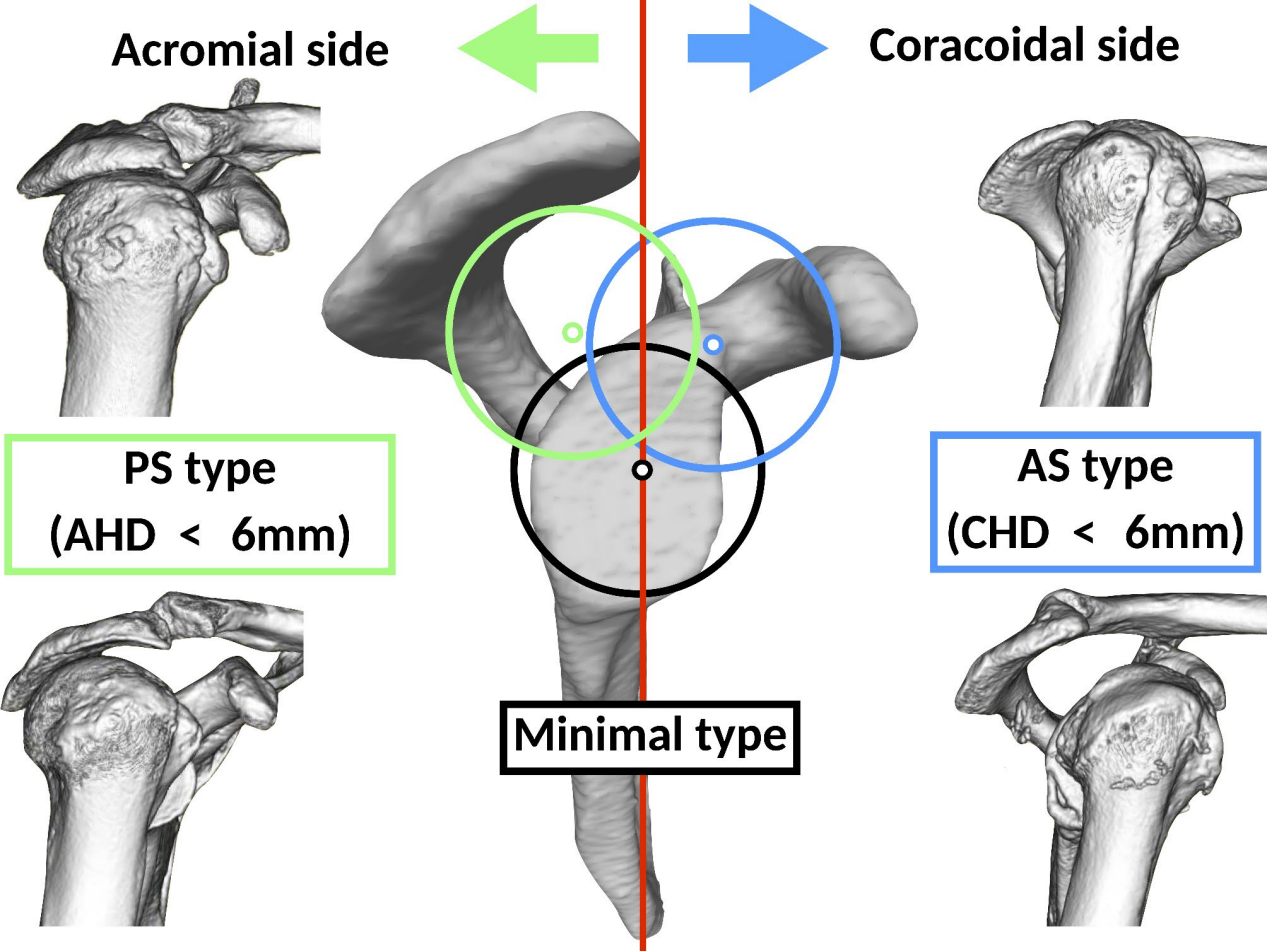
The humeral head translation was categorized into three types based on its position: minimal type (minimal migration), posterosuperior (PS) type (translation towards the acromion), and anterosuperior (AS) type (translation towards the coracoid)

Using preoperative 3DCT, humeral head translation was classified into three types based on its position: minimal type (minimal migration), posterosuperior (PS) type (translation towards the acromion), and anterosuperior (AS) type (translation towards the coracoid) (Fig. 1).

The minimal type was defined as those cases where both the AHD and CHD measurements were greater than or equal to 6 mm. An AHD of 6 mm or greater corresponds to grade 1 in the Hamada classification [2, 16]. Since CHD less than 6 mm is associated with the anterior translation of the humeral head and subscapularis tears [8, 17], we defined 6 mm or greater as the minimal migration.

AHD and CHD measurements were calculated using Meshlab software (version 1.3.3; Institute of Information Science and Technologies, Pisa, Italy) [18]. These distances were measured using 3DCT between the acromion and the humeral head for AHD, and between the coracoid and the humeral head for CHD.

**Fig. 2:**
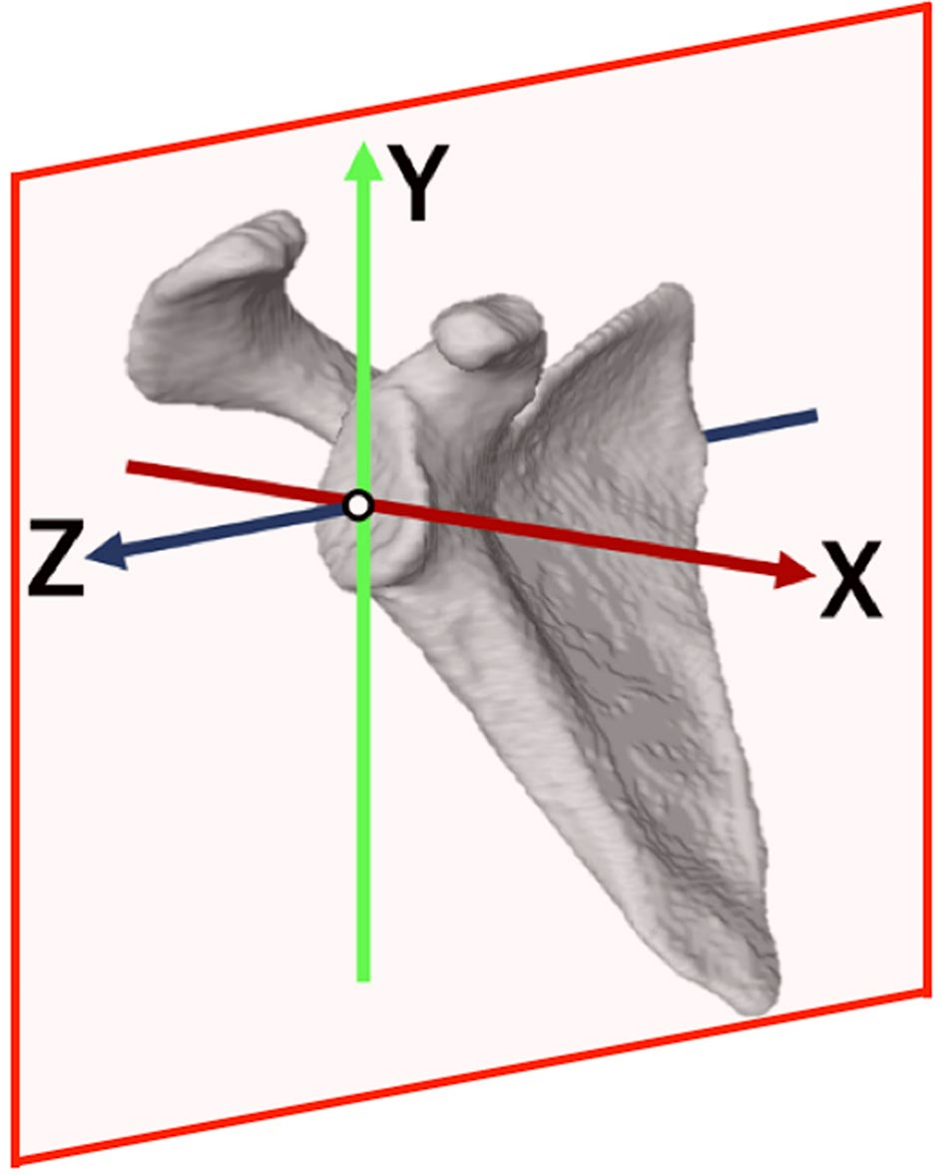
Scapular coordinate system. The plane formed by the Y- and Z-axes was used to evaluate whether the humeral head translation was anterior or posterior. When the center of the humeral head was located anteriorly, the X-axis value was positive; conversely, when it was located posteriorly, the X-axis value was negative

Distances of less than 6 mm in AHD were classified as PS type, while distances of less than 6 mm in CHD were classified as AS type. These measurements indicate a potential rotator cuff tear, as observed in previous studies [2, 6, 8, 19]. Cases in which both the AHD and CHD were less than 6 mm were classified as PS type if the humeral head translated posteriorly from the center of the glenoid cavity, and as AS type if it translated anteriorly. To determine whether the humeral head translated anteriorly or posteriorly, a plane was defined on the glenoid to establish the scapular coordinate system (Fig. 2). The origin was set at point the center of the glenoid cavity, and the scapular coordinate system was constructed in accordance with the International Society of Biomechanics recommendations for all other parameters [20, 21]. The bony landmarks used were the center of the glenoid cavity, the scapular spine, and the inferior angle. The plane formed by the Y- and Z-axes was used as the anterior-posterior boundary. The PS type was defined as cases where the center of the humeral head was located posterior to this plane, while the AS type was defined as cases where it was located anterior to the same plane.

### Acromial and coracoidal spurs

**Fig. 3:**
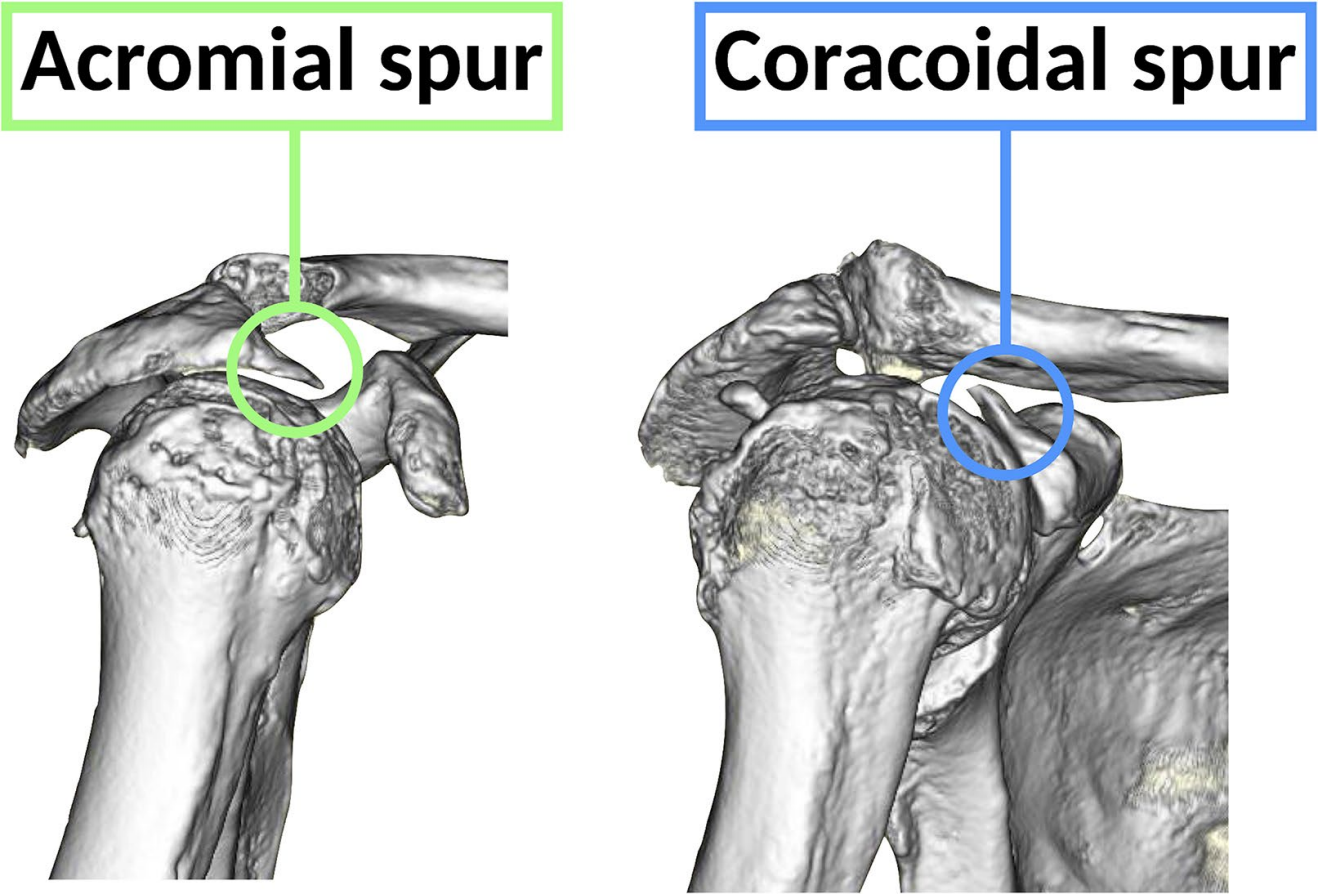
Acromial and coracoidal spurs were evaluated using preoperative 3DCT

The presence or absence of acromial and coracoidal spurs were evaluated using preoperative 3DCT (Fig. 3). If the inclination of the anterior edge of the acromion did not change, we determined that no spur formation was present, even if the acromion had a sharp anteroinferior edge [7]. In contrast, any protrusion on the coracoid was considered spur formation.

### Location of tendon tears

**Fig. 4:**
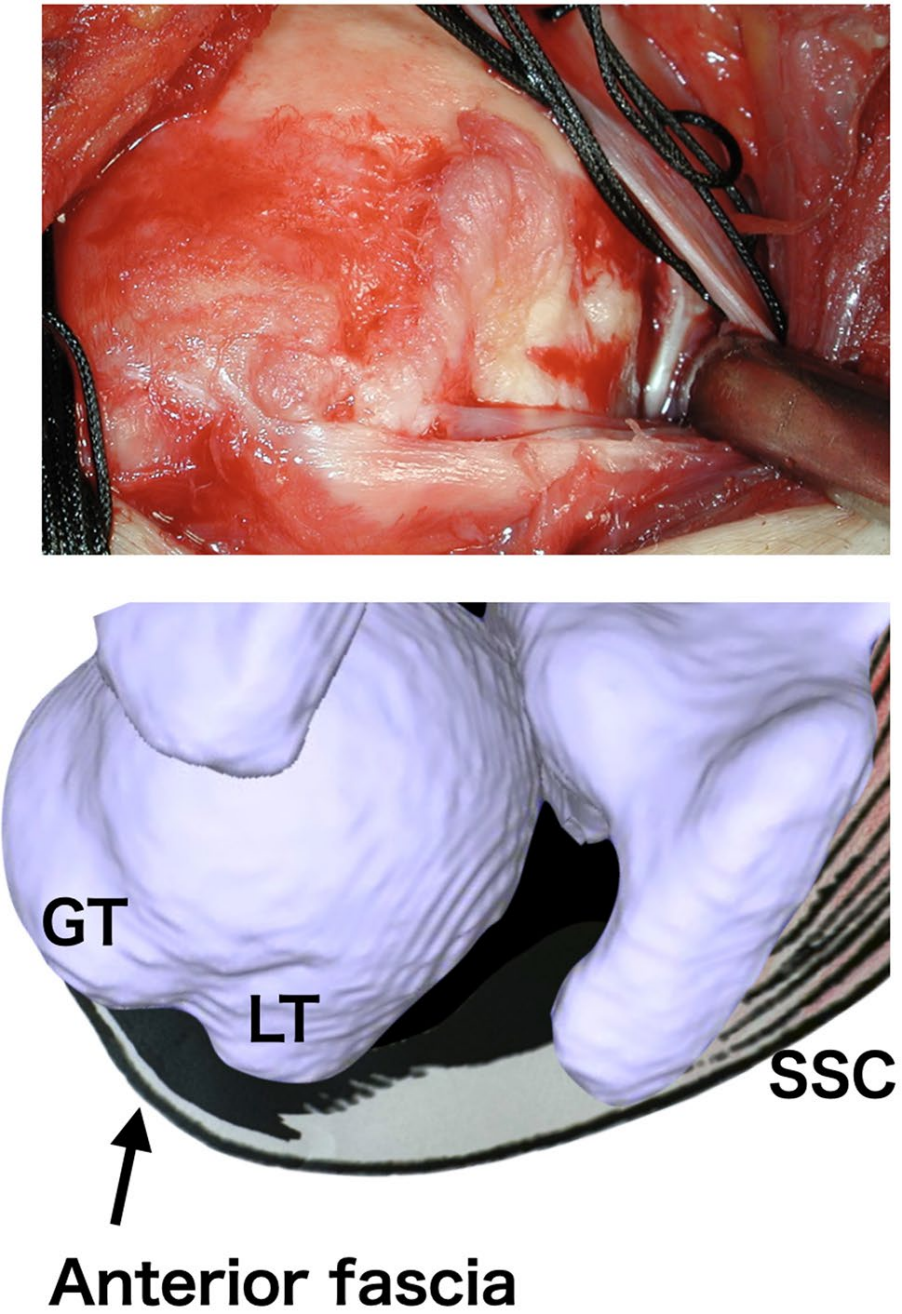
Depiction of the anterior fascia. GT: greater tuberosity; LT: lesser tuberosity; SSC: subscapularis

The presence of tears in the rotator cuff tendons, composed of teres minor (TM), infraspinatus (ISP), supraspinatus (SSP), and subscapularis (SSC), was evaluated through preoperative MRI and intraoperative findings. Additionally, ruptures of the anterior fascia [22] leading to the greater tuberosity covering the surface of the subscapularis (SSC) or the long head of the biceps tendon (LHB) were evaluated using the same methods (Fig. 4).

### Statistical analysis

SPSS Statistics 29.0.0.0 software (IBM Corp., Armonk, NY, USA) was used for the statistical analyses. Image analysis for all cases was conducted by two authors (Y.Y. and Y.A.), and no differences were observed in the classification of humeral head translations, spur formations, and the location of tendon tears. Three nominal variables, including humeral head translations, spur formations, and the location of tendon tears, were evaluated for their relationships using the Chi-square test. Post hoc analysis was performed on the significant Chi-square values by calculating the adjusted standardized residuals (ASR) to identify the cells with an ASR greater than 1.96 or less than − 1.96. Phi and Cramer’s V were calculated to determine the strength of association between these nominal variables. The level of significance was set at *p* < 0.05.

## Results

Humeral head translations were minimal type in 8 cases, PS type in 16 cases, and AS type in 6 cases. Acromial spurs were observed in 22 cases, and coracoidal spurs in 3 cases. Tendon tear locations included the TM in 2 cases, ISP in 29 cases, SSP in all 30 cases, and SSC in 13 cases. Eight cases exhibited anterior fascia ruptures along with SSC tears, and 12 cases showed LHB ruptures, all of which had concurrent SSP and ISP tears.

Regarding the relationship between humeral head translations and spur formations, the PS type showed a significant presence of acromial spurs (ASR = 2.7, *p* = 0.026) and an absence of coracoidal spurs (ASR = -2.0, *p* < 0.001). The AS type showed a significant presence of coracoidal spurs (ASR = 3.7, *p* < 0.001) (Table 1).

**Table 1:** Relationship between humeral head translations and spur formations

	Humeral head translation										
	Minimal type *n* = 8			PS type *n* = 16			AS type *n* = 6			*p* value	Cramer’s V
	n	%	ASR	n	%	ASR	n	%	ASR		
Acromial spur	4	50	-1.7	15	93.8	2.7^*†*^	3	50	-1.4	0.026*	0.494
Coracoidal spur	0	0	-1.1	0	0	-2.0^*†*^	3	50	3.7^*†*^	< 0.001*	0.667

*PS: posterosuperior; AS: anterosuperior; ASR: Adjusted Standardized Residuals;*^*†*^*ASR *> |1.96|; *p < *0.05*

In terms of the relationship between humeral head translations and the location of tendon tears, the AS type had a significant occurrence of the SSC tears (ASR = 3.1, *p* = 0.007), anterior fascia ruptures (ASR = 4.5, *p* < 0.001), and LHB ruptures (ASR = 2.4, *p* = 0.05). The minimal type significantly preserved the anterior fascia (ASR = -2.0, *p* < 0.001) (Table 2).

**Table 2:** Relationship between humeral head translations and location of tendon tears

	Humeral head translation										
	Minimal type *n* = 8			PS type *n* = 16			AS type *n* = 6			*p* value	Cramer’s V
	n	%	ASR	n	%	ASR	n	%	ASR		
TM tear	0	0	-0.9	2	12.5	1.4	0	0	-0.7	0.392	0.25
ISP tear	7	87.5	-1.7	16	100	1.1	6	100	0.5	0.241	0.308
SSP tear	8	100		14	100		6	100			
SSC tear	2	25	-1.2	5	31.3	-1.4	6	100	3.1^*†*^	0.007*	0.574
Anterior fascia rupture	0	0	-2.0^*†*^	2	12.5	-1.9	6	100	4.5^*†*^	< 0.001*	0.838
LHB rupture	2	25	-1.0	5	31.3	-1.0	5	83.3	2.4^*†*^	0.05*	0.446

*TM: teres minor; ISP: infraspinatus; SSP: supraspinatus; SSC: subscapularis; LHB: long head of the biceps tendon; ASR: Adjusted Standardized Residuals;*^*†*^*ASR* > |1.96|; *p < *0.05*

Concerning the relationship between spur formations and the location of tendon tears, coracoidal spurs were significantly associated with SSC tears (*p* = 0.037), anterior fascia ruptures (*p* = 0.002), and LHB ruptures (*p* = 0.025) (Table 3).

**Table 3:** Relationship between spur formations and location of tendon tears

	Acromial spur						Coracoidal spur					
	− *n* = 8		+ *n* = 22		*p* value	Phi	− *n* = 27		+ *n* = 3		*p* value	Phi
	n	%	n	%			n	%	n	%		
TM tear	0	0	2	9.1	0.377	0.161	2	7.4	0	0	0.626	-0.089
ISP tear	8	100	21	95.5	0.540	-0.112	26	96.3	3	100	0.735	0.062
SSP tear	8	100	22	100			27	100	3	100		
SSC tear	4	50	9	40.9	0.657	-0.081	10	37	3	100	0.037*	0.381
Anterior fascia rupture	3	37.5	5	22.7	0.418	-0.148	5	18.5	3	100	0.002*	0.553
LHB rupture	3	37.5	9	40.9	0.866	0.31	9	33.3	3	100	0.025*	0.408

*TM: teres minor; ISP: infraspinatus; SSP: supraspinatus; SSC: subscapularis; LHB: long head of the biceps tendon; +: Spur formation;* −: Without spur formation; *p < *0.05*

## Discussion

This study revealed the relationship between the directions of humeral head translation, the presence of acromial or coracoidal spurs, and the locations of tendon tears in patients with massive rotator cuff tears. Our results showed that in shoulders with massive rotator cuff tears, AS humeral head translation and coracoid spurs indicate SSC tears, anterior fascia ruptures, and LHB ruptures, suggesting an association with anterior instability.

Regarding humeral head translation, both acromial and coracoidal spurs tended to accompany the direction of humeral head translations, suggesting that spur formations are secondary bony changes resulting from humeral head movement. Although narrowing of the AHD implies a rotator cuff tear [3–6], no significant relationships were observed between the PS types and the location of tendon tears, as all cases of massive rotator cuff tears involved SSP tears. Consequently, no significant relationships were observed between the presence of acromial spurs and the location of tendon tears.

We also observed less translation of the humeral head when the anterior fascia was preserved, even in the presence of SSC tears and LHB ruptures. While the anterior fascia may help reduce anterior humeral head translation under these conditions, it is considered to have limited functional significance as it lacks dynamic stability.

In summary, this study examined the relationship between the directions of humeral head translation, the presence of acromial or coracoidal spurs, and the locations of tendon tears in patients with massive rotator cuff tears. The findings suggest that AS humeral head translation and coracoidal spurs are associated with SSC tears, anterior fascia ruptures, and LHB ruptures. It is proposed that in cases of massive rotator cuff tears, the humeral head migrates towards areas where the surrounding tissue has degenerated, leading to repetitive impingement on the adjacent bony structures.

### Limitations

This study had several limitations. The first limitation was that CT scanning was performed in the supine position. It is known that shoulder alignment differs between supine and standing positions [18, 23]; therefore, the findings might vary in the standing position. Second, the evaluation is a static evaluation with their arms lowered. It is known that humeral head translation differs from elevated positions [24] but the effects of dynamic factors have not been evaluated.

## Data Availability

The datasets used and/or analyzed during the current study are available from the corresponding author on reasonable request.

## Abbreviations

AHD: Acromiohumeral distance
ASR: Adjusted standardized residuals
AS: Anterosuperior
CHD: Coracohumeral distance
ISP: Infraspinatus
LHB: Long head of the biceps tendon
MRI: Magnetic resonance imaging
PS: Posterosuperior
SSC: Subscapularis
SSP: Supraspinatus
TM: Teres minor
3DCT: Three-dimensional computed tomography
